# Estimating network changes from lifespan measurements using a parsimonious gene network model of cellular aging

**DOI:** 10.1186/s12859-019-3177-7

**Published:** 2019-11-20

**Authors:** Hong Qin

**Affiliations:** Department of Computer Science and Engineering, Department of Biology, Geology and Environmental Science, SimCenter, University of Tennessee at Chattanooga, Chattanooga, 37403 TN U.S.A.

**Keywords:** Replicative lifespan, Cellular aging, Gompertz, Gene networks, *Saccharomyces cerevisiae*

## Abstract

**Background:**

Cellular aging is best studied in the budding yeast *Saccharomyces cerevisiae*. As an example of a pleiotropic trait, yeast lifespan is influenced by hundreds of interconnected genes. However, no quantitative methods are currently available to infer system-level changes in gene networks during cellular aging.

**Results:**

We propose a parsimonious mathematical model of cellular aging based on stochastic gene interaction networks. This network model is made of only non-aging components: the strength of gene interactions declines with a constant mortality rate. Death of a cell occurs in the model when an essential node loses all of its interactions with other nodes, and is equivalent to the deletion of an essential gene. Stochasticity of gene interactions is modeled using a binomial distribution. We show that the exponential increase of mortality rate over time can emerge from this gene network model during the early stages of aging.We developed a maximal likelihood approach to estimate three lifespan-influencing network parameters from experimental lifespans: *t*_0_, the initial virtual age of the network system; *n*, the average lifespan-influencing interactions per essential node; and *R*, the initial mortality rate. We applied this model to yeast mutants with known effects on replicative lifespans. We found that deletion of *SIR2*, *FOB1*, and *HXK2* considerably altered the initial virtual age but not the average lifespan-influencing interactions per essential node, suggesting that these mutations mainly influence the reliability of gene interactions but not the overall configurations of gene networks.We applied this model to investigate replicative lifespans of yeast natural isolates. We estimated that the average number of lifespan-influencing interactions per essential node is 7.0 (6.1–8) and the average estimated initial virtual age is 45.4 (30.6–74) cell divisions in these isolates. We also found that *t*_0_ could potentially mediate the observed Strehler-Mildvan correlation in yeast natural isolates.

**Conclusions:**

Our theoretical model provides a parsimonious interpretation of experimental lifespan data from the perspective of gene networks. We hope that our work will stimulate more interest in developing network models to study aging as a pleiotropic trait.

## Background

Understanding cellular aging is critical to our understanding of aging in general [1]. At the molecular level, pathways that are known to influence lifespan often play important and conserved functions within cells [2]. Molecular mechanisms of cellular aging are best understood in the budding yeast *Saccharomyces cerevisiae*, a single-cell model organism [3–6]. The lifespan extension effect of sirtuins and the TOR pathways were extensively studied in the budding yeast and were found to be conserved in other species [2, 7–9].

Aging of yeast cells can be measured by the replicative lifespan — the number of cell divisions that cells can accomplish before senescence—and the chronological lifespan — how long cells can retain their proliferative capability in the stationary phase [5]. The replicative lifespan of yeast cells is analogous to the limited replicative capability of primary culture cells that was first observed in human cells [10]. Survival curves of replicatively aged yeast cells are generally sigmoidal and can be described by the Gompertz model [11]. Genome-wide experimental studies have demonstrated changes at gene network levels during the yeast aging process [12]. Cellular aging in yeast is a stochastic process because a population of genotypically homogeneous cells can live to different ages. Broad sense heritability of yeast replicative lifespan has been estimated to be around 22% [11].

In general, aging is quantitatively defined by mortality rate *μ*(*t*), which is the normalized declining rate of viability *S*(*t*):

1 $$\begin{array}{*{20}l} \text{Mortality rate:} \quad & & \mu(t) = - \frac{1}{S(t)} \frac{dS(t)}{dt}, \end{array} $$

where *t* is time. Mortality rate *μ*(*t*) describes the chance of dying over age, and aging occurs when mortality rate is a positive and increasing function of time. Mortality rate is also known as the force of mortality, failure rate, hazard rate, and intensity function in various contexts [13–15]. Mortality rate *μ*(*t*) is often an exponential function of time for biological aging, known as the Gompertz model [16, 17].

2 $$\begin{array}{*{20}l} \text{Gompertz model:} & & \mu (t) = R e^{Gt}. \end{array} $$

In the Gompertz model, *R* is the initial mortality rate when *t* is zero, and *G* is the Gompertz coefficient. The initial mortality rate *R* can be interpreted as the lifespan potential at birth. The Gompertz coefficient *G* has a unit of 1/time, describes the acceleration of mortality rate *μ* over time, and hence is a measure for the rate of aging. Given the role of gene networks in cellular aging, it would be informative to gauge gene network changes during yeast aging. It is not clear how the classical Gompertz model of aging can be used to interpret molecular mechanisms from yeast experimental aging data.

Reliability theory is a well-established field in engineering [14, 15], and its application in biological aging was recognized decades ago [18–24]. Murphy proposed a Bingo model in 1978 and considered an organism as a serial configuration of subsystems [18]. Similarly, Skurnick and Kemeny, in 1978, modeled an organism as a number of serial links and recognized that the weakest link determines the organism’s age [19]. In 1985, Witten argued that an organism can be modeled as a graph and explored ways to regenerate the Gompertz model using a serial configuration of components [20]. Gavrilov and Gavrilova recognized the importance of non-aging components and developed a sophisticated reliability model of aging [23, 24]. All of these previous reliability models are based on serially connected subsystems, analogous to serially connected fuse boxes. These previous models did not capture interaction patterns in molecular networks and, consequently, have not become effective tools to assist molecular studies of aging, a challenge that we aim to address.

The rationale of our modeling approach is based on the need to develop a quantitative framework to evaluate gene network changes during cellular aging. A null hypothesis is often required in statistical analysis and interpretation of experimental results. If experimental data can be sufficiently accounted for by a simple null model, alternative models with more complicated assumptions would not be justified. To provide a quantitative framework to evaluate gene network changes during cellular aging, we propose a parsimonious gene network model that can serve as a null hypothesis. Given the quantitative definition of aging in Eq. 1, a system or an organism can be non-aging when *μ*(*t*) is a constant *C*, which indicates a constant chance of dying over time [23, 24]. In this kind of non-aging organism, the drop of viability is exponential, *S*=*e*^−*Ct*^, and is identical to the exponential decay of radioactive isotopes. Intuitively, as long as non-aging individuals can live to the next day, their chances of survival will be as good as those on the previous day. In bacterial phages, drop of viability is exponential [25], indicative of non-aging characteristics with a Gompertz coefficient *G* of zero. Hence, the null hypothesis for cellular aging must assume that the components of the network systems are non-aging and have constant mortality rates.

In the following sections, we first propose a parsimonious network model for cellular aging, then develop a maximum likelihood approach for parameter estimations, and, finally apply the model to infer global gene network parameters from replicative lifespan data of the budding yeast *Saccharomyces cerevisiae*.

## Model

The first step in developing our gene network model for cellular aging is to model the phenotype of cellular death. We then modified the classical reliability model of aging into a stochastic network model.

### Modeling the phenotype of cellular death

**Fig. 1:**
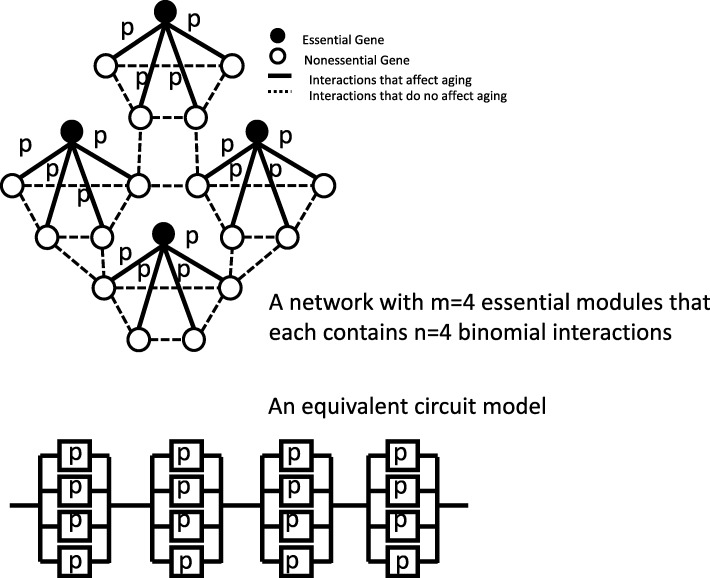
The proposed parsimonious gene network model for cellular aging. We assume that there are *n* number of aging-relevant interactions per essential node and that these interactions are active in cells with a probability of *p* at time zero. There are *m* number of essential nodes in the network. This proposed network model is equivalent to the classical reliability block model

**Fig. 2:**
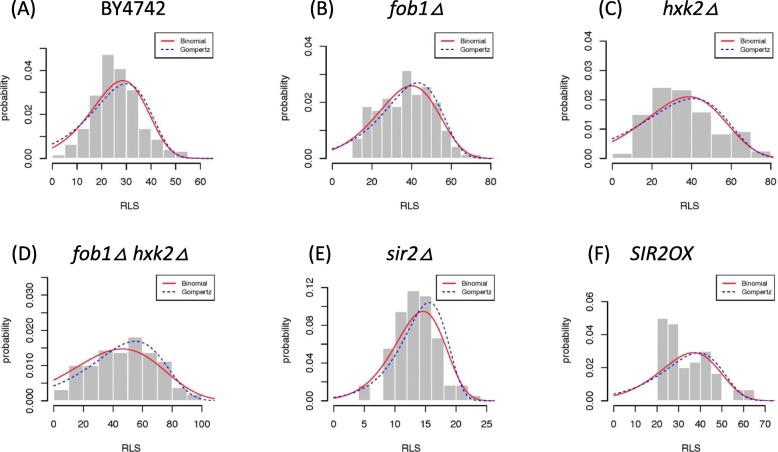
Overlay of fitting curves with lifespan histograms in yeast mutants. Red fitting curves represent the binomial form of the network aging model, and blue fitting curves represent the two-parameter Gompertz model. **a** BY4742. **b** *fob*1*Δ*. **c** *hxk*2*Δ*. **d** *fob*1*Δhxk*2*Δ*. **e** *sir*2*Δ*. **f** *SIR*2*OX*

We introduce the concept of an essential network module, the basic building unit of our network model, to model cellular death as a phenotype. About 17% of the 6600 genes in the yeast genome are essential ones: deletion of any one of these genes leads to inviable cells [26, 27]. We assume that one essential gene or essential node (represented by a solid black circle) interacts with *n* number of non-essential genes/nodes (represented by open circles) in each essential network module (Fig. 1). Based on our parsimonious rationale, gene interactions are assumed to be non-aging: the strength of each gene interaction declines exponentially over time. All gene interactions have the same constant mortality rate *λ* in this model. For clarity, decline in non-aging interactions will be termed decay, and the constant mortality rate will be called the decay rate. An essential node will cease to be active when it loses all of its *n* interactions, a scenario that is equivalent to the deletion of an essential gene, and leads to the failure of the entire network, i.e., cell death. The viability of each non-aging interaction is *e*^−*λt*^. We assume that decaying strength of gene interactions are independent. In other words, loss of one gene interaction will not affect the strength of the remaining gene interactions. This essential network module is equivalent to a circuit block with *n* parallel components in the classical reliability aging model (see Figure 2b in reference [24]). Based on the reliability theory [15, 24], the viability of the essential module is

3 $$ S_{m}(t) = 1 - (1-e^{-\lambda t})^{n} \quad  $$

and the mortality rate of the essential module, *μ*_*m*_ is

4 $$ \mu_{m}(t) = - \frac{dS_{m}(t)}{S_{m}(t) dt} = \frac{n \lambda e^{-\lambda t} (1- e^{-\lambda t})^{n-1} }{1 - (1-e^{-\lambda t})^{n}}\quad.  $$

If we focus on lifespans *t*≪1/*λ*, the above equation can be simplified to

5 $$ \mu_{m}(t) \thickapprox n \lambda^{n} t^{n-1}.  $$

In empirical networks such as yeast protein interaction networks, essential genes/proteins often interact with many other genes. Our model assumes that, among these interactions, only *n* interactions on average are relevant to cellular survival upon deletion of this essential node. In other words, this network model assumes that, on average, there are *n* number of interactions that are relevant to the essentiality for each essential node. We like to emphasize that the proposed exponential change of gene interaction strength is an imperative assumption for a null hypothesis. In other words, we argue that network models with non-exponential changes of gene interactions are alternative hypotheses and should only be used when they offer significantly better fit to experimental data than the null network model with non-aging gene interactions.

### A parsimonious stochastic gene network model for cellular aging

We can now build a stochastic gene network model using the essential network modules. We assume there are *m* number of essential modules to build a network model of aging as in Fig. 1. We assume that failure of any essential module leads to failure of the entire network and, therefore, cell death. This is a reasonable assumption because the absence of any single essential gene leads to inviable yeast cells [27]. We assume that essential genes do not interact with each other and that their failures are independent. With these assumptions, the network model is mathematically equivalent to the serial construction of blocks in the circuit model proposed by Gavrilov and Gavrilova [23, 24].

We assume that gene interactions are stochastic and that the chance of a gene interaction being active is *p* at time *t*=0 (Fig. 1). In intracellular gene networks, gene interactions are inherently stochastic due to the limited number of gene products, noise in protein expressions, and the crowding nature of intracellular spaces [28–30]. Furthermore, transcription noises can be amplified into noises at protein levels [31]. This stochastic network model is mathematically equivalent to the classic circuit model with binomially active components [24]. If a network contains *m* essential modules and each essential gene stochastically interacts with *n* non-essential genes, based on Appendix C in reference [24], the mortality rate of the entire network is

6 $$\begin{array}{*{20}l} \mu_{net} (t) \thickapprox & c m n \lambda p \sum_{i=1}^{n}\binom{n-1}{i-1} (p \lambda t)^{i-1} (1-p)^{(n-1)-(i-1)} \quad,  \\ & \quad \quad \quad \quad \quad \quad \quad \quad \quad \quad \quad \text{when \(t \ll 1/\lambda\)},  \end{array} $$

and where *c* is a normalizing constant, $c = \frac {1}{1-(1-p)^{n}} $. It is reasonable to approximate the modular mortality rate as a summation of possible connection patterns in Eq. 6 if we focus on the range of lifespans *t*≪1/*λ* [24, 32]. The summation term in Eq. 6 is the binomial formula [(1−*p*)+*pλt*]^*n*−1^, which leads to the following re-arrangements:

7 $$\begin{array}{*{20}l} \mu_{net} (t) \thickapprox & cmn (p\lambda)^{n} (\frac{1-p}{p\lambda} +t)^{n-1} \label {n-1 binomial} \end{array} $$

8 $$\begin{array}{*{20}l} = & R (1 + t/t_{0})^{n-1},  \end{array} $$

where

9 $$\begin{array}{*{20}l} t_{0} & = \frac{1-p}{p \lambda} \quad and  \end{array} $$

10 $$\begin{array}{*{20}l} R &= cmn (p \lambda)^{n} t_{0}^{n-1}. \quad \label {Eq R} \end{array} $$

The parameter *t*_0_ has the unit of time and is termed the initial virtual age of the system (IVAS). The parameter *R* is equivalent to the initial mortality rate in the classical Gompertz model [23, 24].

The three-parameter mortality function in Eq. 8 can be used to fit an experimental lifespan data set, which can reveal *t*_0_ (the initial virtual lifespan) and *n* (the number of lifespan-influencing interactions per essential node).

The network survival function based on the mortality function in Eq. 8 is found to be

11 $$ S_{net}(t) = e^{\frac{R t_{0}}{n}(1-(1+t/t_{0})^{n})},  $$

and the probability density function of network aging is found to be

12 $$\begin{array}{*{20}l} f_{net}(t) = S_{net}(t) \cdot \mu_{net}(t). \end{array} $$

The maximum of the log-transformed likelihood summed over the entire experimental data set will yield estimations of model parameters. We have implemented these numerical procedures in R codes.

Given our simple assumptions, it is important to test the utility of this proposed parsimonious model for cellular aging. Hence, we applied this network model of cellular aging to the replicative aging of the budding yeast due to the availability of many experimental data sets obtained under controlled conditions. We suggest that the estimated *n* from experimental lifespan data sets may be termed the apparent average number of lifespan-influencing interactions per essential node.

## Results and discussion

### Application in yeast mutants with known effects on replicative lifespan

To further demonstrate the utility of our proposed model, we applied it to experimental replicative lifespan measurements of yeast mutants with known effects on aging [33]. We estimated model parameters from replicative lifespans using maximum likelihood methods. Replicative lifespans were bootstrapped to mitigate potential ascertainment errors.

*SIR2* is a NAD-dependent deacetylase involved in chromosome silencing, chromosome segregation, and DNA recombination. Deletion of *SIR2* shortens yeast replicative lifespan, and over-expression of *SIR2* extends it [33, 34]. As shown in Table 1, our model fitting results show drastically decreased *t*_0_ estimation in *sir2 Δ* —the deletion mutant of *SIR2* and moderately increased *t*_0_ estimation in *SIR2OX*—the over-expression mutant of *SIR2* in comparison to the wildtype control BY4742. Based on Eq. 9, *t*_0_ is inversely associated with the interaction decaying rate *λ*. Lower values of *λ* indicate stronger reliability of protein interactions. Hence, decreased value of *t*_0_ suggests that deletion of *SIR2* decreases the reliability of gene interactions, whereas over-expression of *SIR2* increases it.

**Table 1:** The *Δ* symbols and lower-cases represent deletion mutations, and *OX* represents over-expression modifications

Strains	Average RLS	Network Model			Gomperz Model		AIC Comparison		
		R	t0	n	Gompertz R	Gompertz G	Weibull AIC	Gompertz AIC	Network AIC
BY4742	26.6±0.566	0.0047±8*e*−04	56.2±10.1	7.9±0.825	0.0066±0.00088	0.09±0.006	1832.25±24.31	1878.29±23.16	1863.53±24.45
*sir*2*Δ*	13.96±0.337	0.0028±5*e*−04	16.6±1.9	8±0.3	0.0035±0.0012	0.29±0.031	483.1±15.47	493.59±15.53	494.34±12.18
*SIR*2*OX*	34.72±1.424	0.0033±7*e*−04	65.2±7.5	7.8±0.514	0.0038±0.00103	0.08±0.009	457.75±8.83	472.71±9.01	471.46±8.46
*fob*1*Δ*	37.72±1.119	0.0032±6*e*−04	71.4±5.7	7.6±0.468	0.0035±0.00068	0.07±0.006	1121.55±13.38	1133.84±13.55	1134.36±12.42
*hxk*2*Δ*	36.77±1.596	0.0057±0.001	101.3±12.9	7.5±0.629	0.0067±0.00109	0.05±0.003	1006.87±13.51	1017.62±9	1016.83±10.47
*fob*1*Δhxk*2*Δ*	48.46±1.565	0.0051±0.0013	118.1±8.3	6.5±0.671	0.0041±0.00063	0.04±0.003	1430.63±12.85	1427.9±11.46	1435.6±13.08

Replicative lifespans of each strain were resampled 100 times using the bootstrap with replacement method. Each resampled lifespan data set was fitted with the binomial network aging model using the maximal likelihood method. The maximal likelihood estimations from fitting to 100 bootstraps were averaged

Mutants of two other yeast genes were also studied. *FOB1* regulates the number of rDNA copies in yeast cells, and its deletion extends yeast replicative lifespan [33]. *HXK2*, a hexokinase, limits glucose input for glycolysis, and its deletion mutant is considered a genetic model for calorie restriction [33]. Our results show that in both single-deletion mutants of *FOB1* and *HXK2*, estimations of *t*_0_ increase and estimations of *n* remain in the same range. In the double-deletion mutants where both *FOB1* and *HXK2* are absent, *t*_0_ increases with the largest mean values, although *n* decreases moderately.

As shown in Table 1, we found that the estimated IVAS *t*_0_ is generally much greater than the average lifespan in these yeast strains. In all the strains studied, the trends of these changes remained when we bootstrapped the experimental measurements, indicating these changes of *t*_0_ are robust to ascertainment fluctuations during replicative lifespan experiments.

When *t*≪*t*_0_, the binomial network mortality rate *μ*_*net*_ will approach the classical two-parameter Gompertz model of aging [24], and the Gompertz coefficient, *G*, is found to be

13 $$ G = \frac{n-1}{t_{0}}. \label {EqG}  $$

Hence, the proposed network model of aging can be viewed as an extension of the two-parameter Gompertz model and provides an alternative model to use in examining cellular aging.

Consistent with our view that the proposed model is an extension of the Gompertz model, the proposed network aging model performs similarly to the Gompertz model during fitting based on the Akaike information criterion (AIC) (Table 1). The ranges of estimated AIC using the network model mostly overlap with those using the Gompertz model. These observations were further supported by the overlay of fitting density curves over the lifespan histograms in these yeast strains (Fig. 2). Generally, when the Gompertz aging model is a good fit for the experimental lifespan—such as for the wildtype BY4742, deletion mutants *sir*2*Δ*,*hxk*2*Δ*, and *fob*1*Δ*—the proposed network aging model is also a reasonably good fit. When lifespan distribution becomes skewed in the over-expression mutant *SIR2OX*, both the Gompertz model and the binomial model become problematic.

To better interpret the estimated network parameters, we compared the estimated network parameters with the protein physical interaction network. The estimated apparent average number of lifespan-influencing interactions per essential node *n* is about 8 in the reference strain BY4742. The median number of protein interactions for essential genes is about 35 per essential gene in the yeast protein physical interaction network obtained from BioGRID (version 3.4.154) [35]. The BioGRID yeast protein physical interacting networks have aggregated many protein interactions measured under many experimental conditions. Our network model of aging considered only pairwise interactions between nodes that are relevant to gene essentiality. These differences may indicate that only a small portion of protein physical interactions are relevant in gene essentiality and the cellular aging process. It is also entirely possible that the binomial analytical form of the proposed network aging model underestimates the average number of aging-relevant interactions per essential node, given the assumptions needed to reach an analytic form of solution. This limitation may be addressed in future studies using simulation approaches to study aging of empirical gene/protein networks.

When applying this simple parsimonious model to analyze experimental data, we suggest that the estimated *n*, termed the apparent average number of lifespan-influencing interactions per essential node, is similar to other theoretical concepts such as the effective population size in population genetics. The effective population size, though often drastically smaller than the apparent size of biological populations, can help us evaluate various models in population genetics. Another example is the effective transmission rate of viruses in epidemiology. In practice, the utility of the proposed network aging model lies in its ability to help gauge potential gene network changes from experimental lifespan results. Consequently, we are currently developing likelihood-based nested model testing approaches to compare the network aging model parameters from different experiments.

Furthermore, our network model offers an interesting perspectives on the aging of bacterial phages [25]. When *G* approaches zero, the value of *t*_0_ approaches infinite based on Eq. 13, which in turn suggests that the value of *λ* approaches zero based on Eq. 9. An extremely small value of *λ*—the decaying rate of gene interaction indicates that the strength of gene interactions can remain strong for a very long time during aging. Hence, our network model predicts that the strength of gene interaction is extremely reliable in bacterial phages.

### Application in yeast wild isolates and implication for the streher-Muldivan correlation

We applied the proposed network model of cellular aging using replicative lifespan data sets of wild isolates of *Saccharomyces cerevisiae* [11]. As shown in Table 2, ranges of AIC values for the network model generally overlap those for the Gompertz model, consistent with our findings using the laboratory strains. We found that the estimated IVAS (*t*_0_) is between 30.6 and 74.0 with a mean value of 45.4 in our collection of wild yeast isolates, which is in the same range of BY4742 (*t*_0_=56.2). The estimated *n* is between 6.1 and 8.0 with a mean value of 7.0, slightly lower than those estimated in the laboratory strain background.

**Table 2:** Application of the network aging model in yeast natural isolates

Strains	Average RLS	Network Model			Gomperz Model		AIC Comparison		
		R	t0	n	Gompertz R	Gompertz G	Weibull AIC	Gompertz AIC	Network AIC
101S	31.46±0.815	0.0025±9*e*−04	36±5.5	6.7±0.697	0.0012±0.00063	0.14±0.024	582.72±16.95	589.2±20.1	608.56±12.37
M1-2	27.9±1.29	0.0034±0.001	40.5±5.3	7.1±0.76	0.0026±0.00117	0.13±0.017	393.19±10.51	389.76±10.11	397.27±6.96
M13	26.6±1.064	0.0034±9*e*−04	40.8±4.2	7.4±0.64	0.003±0.0011	0.12±0.012	520.85±18.13	504±10.2	510.83±8.61
M14	36.32±1.621	0.0035±0.001	55.1±6.6	6.6±0.637	0.0021±0.00093	0.09±0.011	476.9±8.55	470.91±8.86	480.81±6.33
M2-8	24.77±0.733	0.0034±6*e*−04	42.4±4	8±0.109	0.0043±0.00104	0.12±0.011	738.35±14.92	748.01±13.64	746.64±13.4
M22	32.07±1.359	0.0033±0.001	46.3±11.8	6.9±1.508	0.002±0.00073	0.11±0.011	449.76±9.87	447.31±8.14	456.56±6.9
M32	28.15±0.973	0.0027±0.0011	34.3±2.6	7±0.714	0.0016±0.00046	0.15±0.011	402.89±10.32	411.68±8.67	419.48±9.48
M34	27.02±0.997	0.0028±7*e*−04	31.4±3.5	6.7±0.616	0.0013±7*e*−04	0.16±0.018	408.27±14.11	397.81±10.93	411.9±7.43
M5	36.85±0.942	0.0034±6*e*−04	74±7.2	7.8±0.381	0.004±0.00085	0.07±0.007	1321.34±14.32	1343.21±17.23	1339.61±14.58
M8	34.79±0.969	0.0018±3*e*−04	30.7±2.9	6.1±0.207	4*e*−04±0.00018	0.16±0.016	401.29±10.71	404.45±10.6	431.77±5.99
RM112N	44.13±1.751	0.0025±6*e*−04	55.4±6	6.2±0.375	0.0011±0.00047	0.09±0.01	470.48±11.35	466.79±10.27	481.52±6.92
S288c	26.36±1.501	0.0051±0.0017	56.8±12.9	7.9±1.341	0.0062±0.00202	0.09±0.016	309.61±9.31	310.8±8.3	312.08±8.13
SGU57	23.9±1.319	0.0065±0.0021	58±19.7	7.9±1.505	0.0077±0.00234	0.09±0.011	439.51±8.84	435.81±7.11	438.37±7.38
YPS128	35.08±1.125	0.0026±8*e*−04	41.9±3.9	6.5±0.486	0.0011±0.00045	0.12±0.011	507.62±12.55	506.89±11.38	522.34±8.71
YPS163	34.41±0.699	0.0023±5*e*−04	37.3±2.6	6.4±0.428	8*e*−04±0.00023	0.13±0.01	923.64±14.97	922.83±15.07	957.15±10.43

Replicative lifespans of each strain were resampled 100 times using the bootstrap with replacement method. Each resampled lifespan data set was fitted with the proposed network aging model using the maximal likelihood method. The maximal likelihood estimations from fitting to 100 bootstraps were averaged

We found that the assumption of *t*≪1/*λ* can reasonably be met. If we assume activation of gene interaction with *p*=0.7, the range of 1/*λ* is 73–173 cell divisions with a mean of 106. If we assume *p*=0.9, the range of 1/*λ* is 283–666 cell divisions with a mean of 408. The average replicative lifespan of these natural isolates is 31. Hence, these results confirm that the assumption of *t*≪1/*λ* for our modeling approach can be met as long as interaction activation probability *p* is greater than 0.5. In other words, the heterogeneity of the gene network should be moderate. For yeast gene/protein networks with over one thousand essential genes, the condition of *t*≪1/*λ* indicates that when a cell dies at the age of *t* due to a particular weak essential module, the remaining gene interactions remain largely functional.

**Fig. 3:**
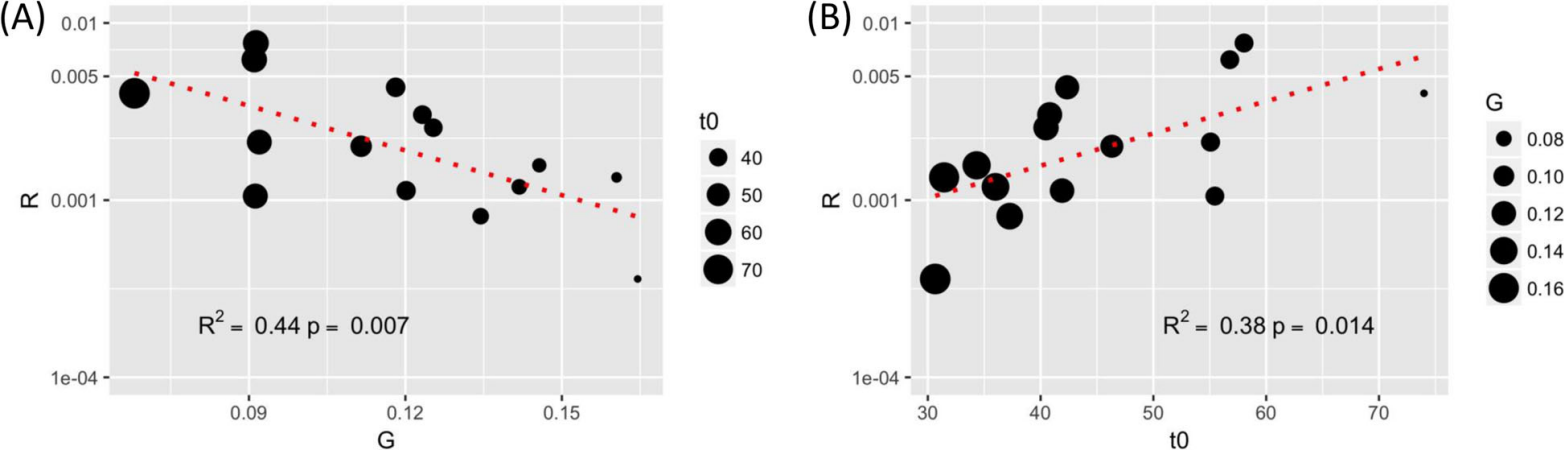
Potential mediator role of *t*_0_ in Strehler-Mildvan correlation in yeast natural isolates. **a** Strehler-Mildvan correlation in studied yeast natural isolates. The size of each data point represents the value of *t*_0_. **b** A positive correlation between *log*_10_*R* and *t*_0_. The size of each data point represents the value of *G*. Mediation tests show that *t*_0_ mediates the correlation between *log*_10_*R* and *G*

The Strehler-Mildvan correlation has led to many studies and debates in the field of research on aging [23, 24, 36–38]. We found this correlation is significant with *p*-value =0.007 and *R*^2^=0.44 (Fig. 3a) in these wild isolates. Interestingly, we found a significant positive correlation between *log*_10_(*R*) and *t*_0_ with *p*-value = 0.014 and *R*^2^ = 0.38 (Fig. 3b). Because of the inverse relationship of *t*_0_ and *G* (see Eq. 13), we tested whether *t*_0_ could mediate the correlation between the two Gompertz parameters *log*_10_(*R*) and *G*. Using the mediation test [39], we found that *t*_0_ mediated 86% of the correlation between *G* and *log*_10_(*R*) with a *p*-value less than 2×10^−16^. The mediation role of *n* was found to be non-significant. These results suggest that *t*_0_ may mediate the Strehler-Mildvan correlation in replicative aging of wild yeasts. It should be noted that there are concerns that the Strehler-Mildvan correlation is caused by a degenerate manifold of Gompertz fit [38]. This degenerate manifold basically leads to a negative auto-correlation between the two Gompertz parameters along a narrow zone of the iso-average-lifespan curve during numerical fitting of homogeneous populations. We addressed these kinds of potential caveats of numerical fitting in one of our previous studies [11] and in a recent study [40]. Because we are dealing with heterogenous yeast cell populations with diverse genotypes, we think our observed Strehler-Mildvan correlation is not caused by the numerical fitting process. We plan to conduct future studies with larger data sets, systematic simulations, and more sophistical mathematical models to fully address these concerns.

The Strehler-Mildvan correlation has led to many studies and debates in the field of research on aging [23, 24, 36–38]. We found this correlation is significant with *p*-value =0.007 and *R*^2^=0.44 (Fig. 3a) in these wild isolates. Interestingly, we found a significant positive correlation between *log*_10_(*R*) and *t*_0_ with *p*-value = 0.014 and *R*^2^ = 0.38 (Fig. 3b). Because of the inverse relationship of *t*_0_ and *G* (see Eq. 13), we tested whether *t*_0_ could mediate the correlation between the two Gompertz parameters *log*_10_(*R*) and *G*. Using the mediation test [39], we found that *t*_0_ mediated 86% of the correlation between *G* and *log*_10_(*R*) with a *p*-value less than 2×10^−16^. The mediation role of *n* was found to be non-significant. These results suggest that *t*_0_ may mediate the Strehler-Mildvan correlation in replicative aging of wild yeasts. It should be noted that there are concerns that the Strehler-Mildvan correlation is caused by a degenerate manifold of Gompertz fit [38]. This degenerate manifold basically leads to a negative auto-correlation between the two Gompertz parameters along a narrow zone of the iso-average-lifespan curve during numerical fitting of homogeneous populations. We addressed these kinds of potential caveats of numerical fitting in one of our previous studies [11] and in a recent study [40]. Because we are dealing with heterogenous yeast cell populations with diverse genotypes, we think our observed Strehler-Mildvan correlation is not caused by the numerical fitting process. We plan to conduct future studies with larger data sets, systematic simulations, and more sophistical mathematical models to fully address these concerns.

## Conclusions

We present a probabilistic gene network model of cellular aging that can serve as a parsimonious model for interpreting experimental lifespan measurement. Our network aging model converts the classic Gompertz coefficient into two parameters: *n* (the average number of lifespan-influencing interactions per essential node) and *t*_0_ (the initial virtual age). The parameter *n* is informative regarding network configuration, and the parameter *t*_0_ is informative regarding interaction reliability and network heterogeneity. Applications of our model in yeast aging showed that our model is as applicable as the classical two-parameter Gompertz model. Overall, we showed that the proposed network aging model can assist with the molecular study of cellular aging. Given the pleiotropic nature of aging, we hope that this work can stimulate more interest in developing more sophisticated network models for the study of aging.

## Data Availability

The data sets and codes are available at https://github.com/hongqin/network_aging_codes_2018.

## Abbreviations

AIC: Akaike information criterion
IVAS: Initial virtual age of the system
